# How Do Age-Group Triathlon Coaches Manage Training Load? A Pilot Study

**DOI:** 10.3390/sports12090261

**Published:** 2024-09-20

**Authors:** David Procida, Jocelyn Mara, Lachlan Mitchell, Naroa Etxebarria

**Affiliations:** 1 Discipline of Sport and Exercise Science, University of Canberra Research Institute of Sport and Exercise, Canberra 2617, Australia; david@procida.com.au (D.P.); jocelyn.mara@canberra.edu.au (J.M.); 2 Victorian Institute of Sport, Melbourne 3206, Australia; lachlan.mitchell@vis.org.au

**Keywords:** multidisciplinary sports, swimming, running, cycling, training management, load quantification, coach behavior

## Abstract

Multidisciplinary sports like triathlons require combining training for three different sports, and it is unclear how triathlon coaches manage this. During a 10-week period, we provided four age-group triathlon coaches with summary reports of the training completed by their athletes (n = 10) in the previous week. Coaches were then asked if the information provided to them was used to inform training prescription for the following week. The information provided to coaches included relative acute training load (rATL) and training stress scores (TSSs). Weekly fluctuations in rATL of >10% (spikes) were 83% (swim), 74% (bike) and 87% (run). Coaches adapted training loads for the upcoming week in 25% of all rATLs reported, and only 5% (swim), 33% (bike) and 9% (run) of the adjusted loads avoided spikes. Consequently, there were 22 single-discipline acute training load spikes vs. 14 spikes when combining all three disciplines. Only 1.5% of training was lost to injury, mostly after a large running-based training load spike (>30%). Coaches largely overlooked the information provided in the report when prescribing exercise for the following week, and when adjusted, it failed to bring weekly load variability <10%.

## 1. Introduction

Multidisciplinary sports such as triathlon require intentional planning of weekly training sessions for three different sports [1,2]. To be adequately prepared for endurance events of this nature, the volume of training is significant [3]; hence, monitoring and reviewing training loads is crucial to enhance training adaptations and consequent performance [4,5,6]. Enhancing training adaptations requires combining adequate types, intensity levels, and volumes of training stimuli interspersed with effective recovery periods [7]. However, prescribed external (objective) training loads might differ from the loads athletes complete [8]; hence, coaches should consider completed workloads by athletes instead of relying on original exercise prescription when planning future training loads.

Suboptimal training preparation may result in compromised performance outcomes for athletes, but perhaps in age-group triathletes, injury prevention comes before performance optimization. Triathlon studies have characterized training undertaken by age-group triathletes [9,10]. Inappropriate training loads may result in injury, illness, or non-functional over-reaching [11,12,13], with injury reported to affect between 29 and 91% of adult triathletes at some stage during training and competition [14]. Injury results in a loss of training consistency, which has a negative impact on individual athletic success [15]. Although no single marker of an athlete’s response to training load consistently predicts maladaptation or injury [16], avoiding abrupt increases or decreases in training load seems to be key to avoid them [17,18]. This is why there are numerous ways to monitor load in endurance sports, such as Training Impulse (TRIMP) [19], training stress scores (TSSs) [20], and relative acute training load (rATL) [21] as well as acute/chronic workload ratio (ACWR) [22]. Most training-load-related outcome measures require context around them and have their limitations in explaining training load; hence, they should be interpreted carefully. Given that age-group or amateur triathletes would comprise a very high portion of registered triathletes world-wide (compared to professional or elite athletes), their training load management and enhancement warrant further research.

In order to identify exercise programming aspects that might negatively affect triathletes, it is crucial to capture training-related data consistently and accurately. The increase in readily available wearable technologies in combination with the growth of online-based athlete monitoring systems make capturing training data a reasonably easy venture. However, despite the ability to capture exercise training metrics such as running distance, intensity of exercise, power output in cycling, etc., it is unclear how this information may be used by coaches to amend or enhance future training prescription, hence the urgency to expand on the understanding and education of coach knowledge and practices on coach behavior in relation to training load management.

It is unclear how triathlon training data are used to inform future exercise prescription in an attempt to align with fundamental principles of training metrics relating to the volume, intensity, frequency, and type of exercise. However, evidence from other sports suggests that sudden increases in training load can increase the likelihood of injury. Such sports include team sports [21,23], as well as individual endurance sports including swimming [24] and running [25]. Coach behavior regarding weekly training load fluctuations would provide insights into how age-group triathlon coaches manage athlete training loads in triathlon, both for each individual discipline and also the combined overall weekly load distribution. Therefore, the primary aim of this study was to investigate if the exercise training load prescribed by triathlon coaches is informed by or adjusted according to the training load undertaken in the previous week(s) or not, in order to avoid substantial fluctuations in training load.

## 2. Materials and Methods

### 2.1. Design

This exploratory observational study focused on a 10-week training data collection period (competition season, May–September) where the training loads of a cohort of age-group athletes were monitored. Coaches were provided with weekly summary reports with the completed loads by athletes in the previous week and were asked whether they used the information provided on training load monitoring metrics to inform the training plan for the following week. All athletes performed standardized time trial-based field testing in all three disciplines (swim, bike and run) prior to commencing the study to determine their basal individual threshold intensities. These data were required to calculate individualized training loads derived from the various relative training intensities.

This study was approved by the Committee for Ethics in Human Research at the University of Canberra (project ID 2030) and all participants provided their written informed consent prior to participating in the study.

### 2.2. Participants

In total, 10 (n = 10) age-group triathletes (females, n = 6; males, n = 4) and 4 (n = 4) male nationally accredited age-group coaches were recruited via a post on social media to participate in this study. The triathletes were 38 ± 6 years old (mean ± SD) and the coaches were 46 ± 8 years old. The coaches had 3.0 ± 1.6 years of experience in coaching age-group triathletes with 13 ± 6 h of weekly commitment. Three coaches had a triathlon development coach accreditation (Level II out of III, AusTriathlon, Milton, QLD, Australia). One coach had an equivalent accreditation as a running coach but coached all three disciplines of triathlon. All coaches would be considered non-elite development coaches. Coaches had 11 ± 6 years of personal triathlon competition experience at the age-group level. All coaches used TrainingPeaks™ (Boulder, CO, USA) as their athlete monitoring system and had 2.3 ± 1.2 years of experience using this system. Coaches coached their athletes face-to-face and also remotely. All participating triathletes were coached by one of the participant coaches. The triathletes had 1 to 4 years of triathlon training experience at age-group competition level. Athletes had completed a wide range of race distances, predominantly Sprint and Olympic distance, and one of them had completed a Half-Ironman. Triathletes had to be healthy when the field-based testing took place and have been training for triathlon races for at least 12 months.

### 2.3. Data Collection

All the athletes used Garmin GPS-enabled smart devices to record training data across the swim, bike, and run. All sessions recorded training time, distance, and pace/speed. For the bike and run sessions, heart rate was recorded using Garmin chest strap-based technology. All data were uploaded to the online athlete monitoring system TrainingPeaks™. The participant coaches were required to use the online platform TrainingPeaks™ as their athlete monitoring system [11], which conveniently provides the training load data required for this study. All athletes performed time trial-based field testing in all three disciplines (swim, bike, and run) prior to commencing the study to determine their basal individual threshold intensities. The field testing consisted of a 1000 m time trial (TT) for swimming, a 30 min TT for the bike (flat open road), and a 30 min TT for the run. These time trials were common field-based tests undertaken by the athletes during a season to monitor progress. Since we were interested in looking at chronic training load and acute training load, we only needed the threshold so that we could determine the intensity factor for each session. The intensity factor and the session duration were then used to calculate the training stress score, which was used for the CTL and ATL. These data were required to calculate individualized training loads derived from the various relative training intensities.

Coaches were provided with several familiarization sessions with the lead researcher (DP) prior to the commencement of the study. The lead researcher provided the coaches with mentoring about all the data collection and outcome measures involved in the study. Coaches completed a questionnaire at the start of the study which included information on the coaches’ background (experience, weekly commitments to coaching, coaching delivery modes), certifications and education relating to coaching, and athletic history. The athletes completed their questionnaire at the start of the study and included information on training and competition experience and a brief injury history summary.

All athlete training data were gathered via the athlete’s smart training devices and the associated sensor and uploaded to TrainingPeaks™ via the athlete’s smartphone application. At the end of each week, the lead researcher accessed the following information: total number of sessions (and per discipline), training session duration, intensity of training, training stress score (TSS), chronic training load, and acute training load for each discipline. The lead researcher then provided the coaches with a summary feedback report for each individual athlete on a Sunday. The next day on the Monday, coaches released the training program for the following week. The summary feedback provided by the research team to the coaches included (i) the weekly average intensity factor (*mean percentage of threshold intensity for a given session,* e.g., *a session completed at 65% of threshold intensity would have an IF of 0.65*); (ii) the training stress score (*IF^2^ × Volume (hours) × 100*), [26] representing the total training load for the week; (iii) the number of sessions planned by the coach; (iv) the number of sessions completed by the athlete; (v) chronic training load: exponentially weighted moving average of the training stress score over the previous 42 days; (vi) acute training load: (vii) exponentially weighted moving average of the training stress score over the previous 7 days; (viii) relative acute training load (rATL): difference in acute training load between the current week and the previous week (%), used to calculate weekly variability in training load; and (ix) acute/chronic workload ratio (ACWR). Although the role of ACWR as a risk injury predictor is contested [27], it was used as a complementary measure to monitor training load overtime.

Given the 42-day period used to calculate chronic load (for each discipline as well as for all three disciplines combined), the threshold testing was performed 42 days prior to the collection of any training load data for the study. During this period, athletes continued training as per usual. The results of both training load metrics for the previous week were color-coded green if they were within the pre-defined range (<10% in rATL, ACWR between 0.8 and 1.5 [28], and compliance between 80 and 110%). When the difference fell outside of these ranges, the metrics were color-coded red. We defined a >10% change in week-to-week training load fluctuations in rATL to be enough to consider it a spike.

Training compliance was monitored as the percentage of prescribed sessions completed by the athlete in the previous week. Finally, the coaches were asked to provide a binary response to the question “Did you use the rATL/ACWR feedback metric to alter training load in the subsequent week?” This information was used to determine whether the coaches considered the training load metrics from the previous week when prescribing their athletes’ training loads for the following week. In instances where the coach indicated that the feedback metrics were not used to adjust the training load, the coaches were asked to provide a reason for it (open text).

### 2.4. Data Analysis

Data analysis was conducted using R version 4.01 in RStudio (version 1.3.959, RStudio Inc., Boston, MA, USA). Descriptive statistics are reported as mean ± SD, unless otherwise stated. In addition, training load and training load prescription were highly individualized and achieved large inter-athlete variation. Therefore, we provided ‘per athlete’ and ‘per coach’ descriptive statistics to present individual athlete data. In instances where the coach indicated that the feedback metrics were not used to adjust the training load, the coaches were asked to provide a reason as a short response. These responses were summarized into codes, which were then used to determine themes using thematic analysis [29] to understand coach behavior regarding training load management.

## 3. Results

### 3.1. Training Load

A total of 770 training sessions were prescribed by the coaches over 10 weeks, and 640 training sessions (663 h of training) were completed by the participant athletes. Out of all the training sessions, only three athletes missed training sessions due to injury or illness, twelve sessions due to injury and fourteen sessions due to illness. Ten of the sessions lost to injury relate to the same athlete and after a rATL of >30% (running-related).

There were no missing data or non-responses from the coaches. Cycle training consistency was highest with 88 ± 36% of sessions being completed, followed by run training (84 ± 29%) and swim training (81 ± 35%). Overall training compliance was 84 ± 25%. A summary of the overall training volume, intensity, and total stress scores for the training sessions during the 10-week period is shown in Table 1.

**Figure 1:**
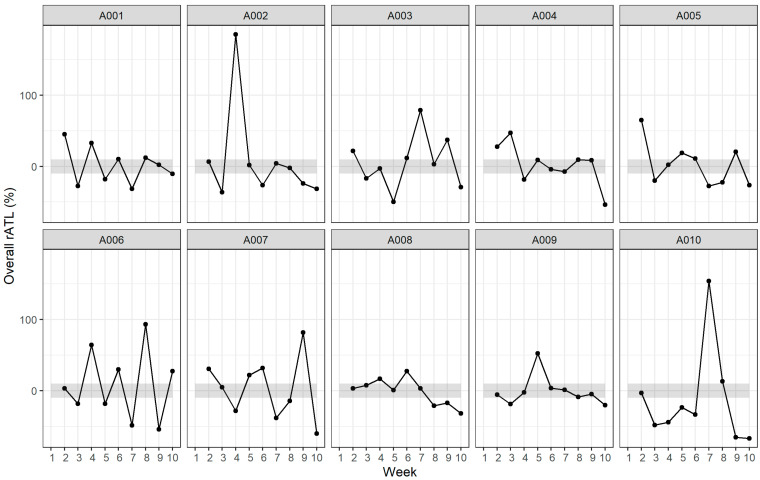
Overall rATL per athlete. A00× indicates the athlete identification number. Gray shaded bands represent ±10% relative acute training load (rATL) range. Symbols indicate whether the coach for each athlete modified the training load based on the rATL feedback metric provided: ‘●’ = feedback was used.

**Figure 2:**
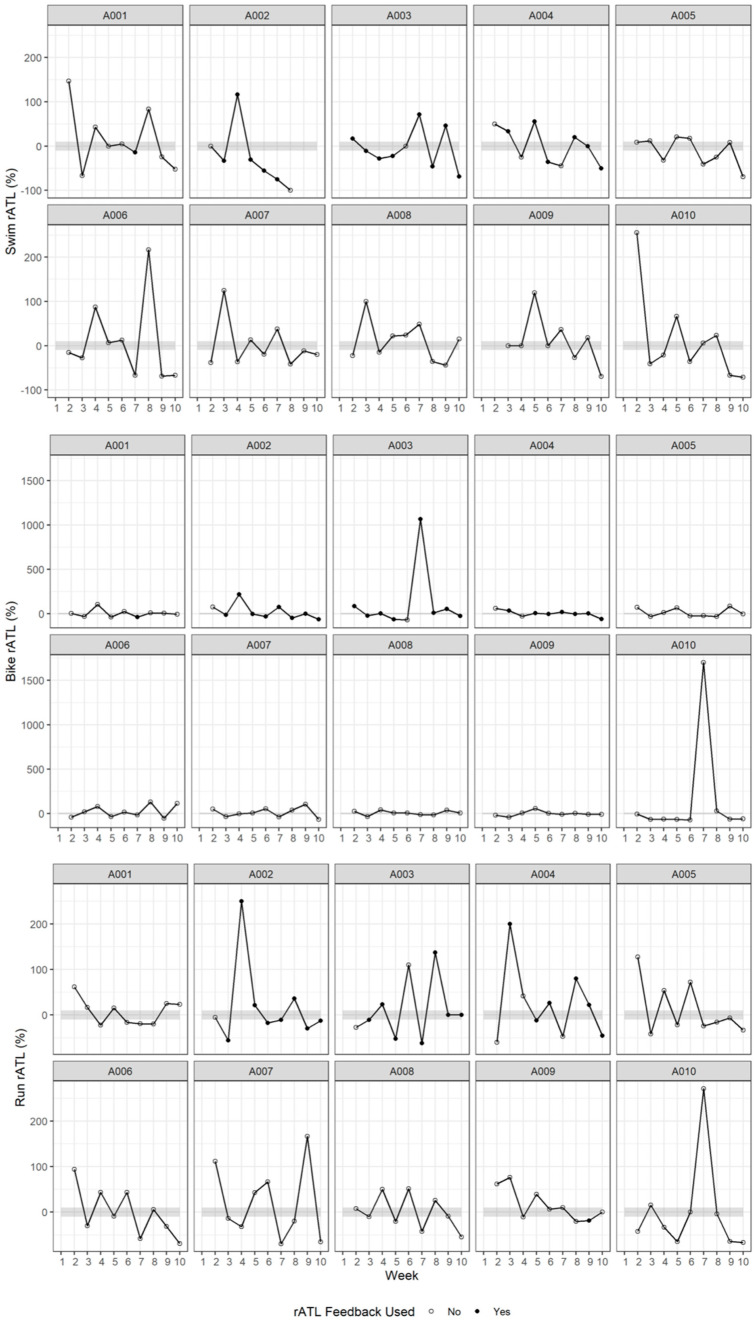
Swim, bike, and run rATL per athlete. A00× indicates the athlete identification number. Gray shaded bands represent ±10% relative acute training load (rATL) range. Symbols indicate whether the coach for each athlete modified the training load based on the rATL feedback metric provided:‘○’ = feedback was not used, ‘●’ = feedback was used.

For the purpose of this study, rATL and ACWR metrics were only used as signposts to guide the training load completed by the athletes (as opposed to predictive outcomes). The weekly training load fluctuations for the 90 person-weeks, each with 3 separate rATLs (1 per discipline), were recorded, with a total of 270 week-to-week fluctuations in rATLs recorded, showing that 71% of them were >10% (Figure 1). Weekly variations in rATL of >10% were 83% for swimming, 74% for cycling, and 87% for running. Moreover, there was a substantial number of weekly fluctuations in rATL of >30% in training load (52% for swimming, 53% for cycling, and 51% for running). There were 32% of weekly fluctuations in rATL >30% for overall training load. All rATLs for each individual athlete for the 10-week data collection period for each discipline (swim, bike, and run) are shown in Figure 2. Coaches considered 23%, 27%, and 24% of the swim, bike, and run (respectively) of all rATL summary feedback reports provided to them (Table 2).

### 3.2. Weekly Reports

From the summary feedback reports where rATL was considered, only 5%, 33%, and 9% of swim, bike, and run rATLs fell within the ±10% variability range the following week. Only 48% (swim), 67% (bike), and 57% (run) of the ACWRs fell between the recommended 0.8 and 1.5, and coaches considered 20% (swim), 23% (bike), and 21% (run) of the ACWR information provided. From the summary feedback reports where ACWR was considered, only 33%, 50%, and 59% of swim, bike, and run rATLs fell within the recommended 0.8 and 1.5. The majority (95%) of the weekly reports considered came from the same coach, who had a higher education degree (i.e., Sport and Recreation with a Coaching Major) and was the most experienced (>5 years). Only 5% of the reports were considered by the others with less experience (1–3 years).

### 3.3. Coach Behavior

The main reason, in 66% of cases, for coaches to not consider the summary report was because they wanted to avoid changing the training they had already planned. The second reason for not considering the summary report was related to environmental constraint (18%), where the training environment impacted the ability of the athlete to train. Illness/injury and work/life constraints combined explained 13% of the cases to not consider the feedback report. Coaches gave the athlete the freedom to decide on training load for that week in 3% of cases.

## 4. Discussion

This exploratory study reports a high incidence of large (10–30%) and ad hoc weekly training load fluctuations in the training prescription of amateur (age-group) triathletes. When faced with the use of an externally provided summary of training metrics (that coaches already have access to) with added interpretation, coaches in most cases did not consider the information provided and relied instead on pre-existing planning for future load prescription. It is unclear whether capturing and processing other information on the weekly reports would have been more valuable for the coaches. High weekly training variation did not result in a high incidence of injuries. It is unclear how effective this training prescription approach would be when the priority is to enhance training adaptations.

There were larger fluctuations in weekly training load metrics when training load was observed separately for each discipline than when the training load of the three disciplines was combined. This outcome underpins the importance of studying each individual discipline separately as well as considering the combined load. The weight-bearing nature of running probably contributed to how most of the injuries in this study were linked to weekly running load fluctuations of >30%, in agreement with previous research [25]. Running results in higher musculoskeletal load compared to the non-weight-bearing exercise like swimming and cycling, and most injuries in triathlon result from it [30]. Running-related training load would be the priority for triathlon coaches to monitor and alter, if needed, in order to minimize injuries. Endurance sports exercise prescription is dominated by remote coaching practices and digital technology [31]. Training management systems such as TrainingPeaks™ allow coaches to amend sessions remotely with ease. Coaches might need to be responsive to adjustments required in training loads at short notice, especially with running, to ensure a progressive and incremental model for training and avoid abrupt spikes.

The weekly training volume undertaken by the triathletes in this study is similar to that described in the literature for recreational triathletes [9]. Athletes and coaches understandably push close to the limits of tolerance of the given athlete to promote performance gains without compromising good health and the risk of injury. Suboptimal training stimuli may preserve good health but might not produce the best performance, while a more aggressive approach with training load might jeopardize the athlete’s health.

Consistency in training has been shown to be a key factor in athletic success [15], where training load is progressive and incremental, fundamentally, despite many different ways to periodize endurance training. Despite the low illness/injury incidence registered over the relatively short 10-week period, with over half of the weekly load fluctuations being >30% across all three single disciplines, it is unclear if the training process is adequate for enhancing adaptations [16]. A combination of failure to consider recently completed workloads by athletes to inform training prescription for the coming week, and athlete compliance factors (i.e., adherence to the prescribed exercise intensity during training sessions), likely contributed to large fluctuations in training load. These two factors are key when athletes and coaches work remotely, and should be the focus when athletes and coaches discuss strategies for training and competition.

Most coaches favored the already-planned workload for the upcoming week without checking the work completed by the athletes in the previous week. Perhaps this decision making is related to the metrics provided to them in the weekly summary reports, or perhaps it is because of coaches expecting/assuming athletes to complete exactly the load prescribed. It could also be that coaches in this study did not have a problem with substantial week-to-week load variations, and they perceive this to not have any negative consequences. These findings in themselves are very interesting, provided that there is limited evidence on the concept of how the exercise prescribed by coaches and the exercise completed by athletes align. Little is understood about how coaches go about exercise prescription and whether the completed exercise by the athletes reflects the exercise prescribed by the coach.

We acknowledge that this exploratory study has a limited number of coach and athlete observations, and provided that there are very limited data on how triathlon coaches use training data to inform future prescription, this study provides some preliminary insights and would encourage future research to include more coaches and athletes in their study designs. Although the present study is limited by a low number of observations, it raises some important and fundamental questions that future research should aim to address, for example, identifying the factors that influence coaches’ training load management decisions in multi-sport environments. The discrepancy or alignment between training load prescription by coaches and training load completion by athletes, not only in terms of sessions completed or not but also in relation to the intensity of the work undertaken (vs. prescribed), is a topic that requires investigating. This knowledge will assist in evaluating the importance of acting on work undertaken instead of relying on work that was planned to inform future exercise prescription.

The practical implications for age-group triathlon coaches include the following:

- Rapid changes (increases) in weekly training load derived from individual disciplines (swimming, cycling, running) might be masked when observed as the overall training load combining all disciplines (swim, cycle and run) compared with the training load of isolated disciplines.
- Running-related workloads are to be monitored more carefully, as the injuries experienced by athletes in this study occurred mostly after a large increase (>30%) in running load.
- Identifying a system to increase responsiveness to adjust future workloads based on previous work undertaken might be advantageous in avoiding large weekly fluctuations that might negatively affect some athletes.

## 5. Conclusions

This study provides preliminary insights into how variable training prescription by coaches and corresponding training load completed by athletes is in age-group triathlons. Coaches were more likely to adhere to pre-determined training loads rather than modifying them based on completed training load feedback metrics, even at the expense of exposing athletes to high training load differentials from one week to the next. The incidence of injuries was mostly related to large weekly fluctuations in running training load, but not all athletes were affected by it, suggesting there is substantial inter-individual variability. Moreover, even when the feedback provided was considered, this did not always avoid a spike in training load (both single-discipline and combined training loads) the following week. This pilot study indicates that the outcome measures chosen in this study might not have been useful for coaches, that coaches might not always seek to avoid large weekly training load fluctuations, and/or that coaches might prescribe training loads that athletes might not adhere to or follow accurately.

## Figures and Tables

**Table 1:** Training load data over the 10-week data collection period (mean ± SD).

Discipline	TSS	IF (%)	Volume (h)
Swim	128 ± 118	85 ± 37	1.3 ± 1.0
Bike	215 ± 161	70 ± 25	3.6 ± 2.6
Run	168 ± 91	83 ± 22	2.2 ± 1.2
Overall	507 ± 245	86 ± 15	6.6 ± 3.1

IF = intensity factor (mean percentage of threshold intensity for a given session, e.g., a session completed at 65% of threshold intensity would have an IF of 0.65); TSS = training stress score (IF^2^ × volume (hours) × 100).

**Table 2:** Number of weeks and athletes each coach was responsible for and the corresponding number of weeks where coaches considered the relative acute training load (rATL) to adapt their training load prescribed to the athletes for the following week; 100% of rATLs considered were calculated as follows: # of athletes X # of weeks × 3 (disciplines). Key: # = number.

rATL Considered by Coaches (# of Weeks)						
Coaches	# of Athletes	# of Athletes × 10 (# of Weeks)	Swim	Bike	Run	% of rATL Considered
C1	2	20	0	0	1	2
C2	3	30	20	23	21	58
C3	1	10	1	1	0	7
C4	4	40	0	0	0	0
Total	10	100	21	24	22	22

## Data Availability

The data are not publicly available due to privacy and ethical restrictions.

## References

[1] Millet G.P., Vleck V.E., Bentley D.J. (2011). Physiological requirements in triathlon. J. Hum. Sport. Exerc..

[2] Mujika I. (2014). Olympic preparation of a world-class female triathlete. Int. J. Sport. Physiol. Perform..

[3] Vleck V., Millet G.P., Alves F.B. (2014). The impact of triathlon training and racing on athletes’ general health. Sport. Med..

[4] Etxebarria N., Mujika I., Pyne D.B. (2019). Training and competition readiness in triathlon. Sports.

[5] Mujika I. (2017). Quantification of Training and Competition Loads in Endurance Sports: Methods and Applications. Int. J. Sport. Physiol. Perform..

[6] Seiler S. (2010). What is best practice for training intensity and duration distribution in endurance athletes?. Int. J. Sport. Physiol. Perform..

[7] Jakovlev N. (1977). Sportbiochemie.

[8] Foster C., Florhaug J.A., Franklin J., Gottschall L., Hrovatin L.A., Parker S., Doleshal P., Dodge C. (2001). A new approach to monitoring exercise training. J. Strength. Cond. Res..

[9] Falk Neto J.H., Parent E.C., Vleck V., Kennedy M.D. (2021). The Training Characteristics of Recreational-Level Triathletes: Influence on Fatigue and Health. Sports.

[10] Vleck V., Massuca L.M., de Moraes R., Falk Neto J.H., Quagliarotti C., Piacentini M.F. (2023). Work, Training and Life Stress in ITU World Olympic Distance Age-Group Championship Triathletes. Sports.

[11] Halson S.L. (2014). Monitoring training load to understand fatigue in athletes. Sport. Med..

[12] Foster C. (1998). Monitoring training in athletes with reference to overtraining syndrome. Med. Sci. Sport. Exerc..

[13] Burns J., Keenan A.M., Redmond A.C. (2003). Factors associated with triathlon-related overuse injuries. J. Orthop. Sport. Phys. Ther..

[14] Vleck V., Millet G.P., Alves F.B. (2013). Triathlon Injury—An update. Schweiz. Z. Med. Traumatol..

[15] Drew M.K., Raysmith B.P., Charlton P.C. (2017). Injuries impair the chance of successful performance by sportspeople: A systematic review. Br. J. Sport. Med..

[16] Borresen J., Lambert M.I. (2009). The quantification of training load, the training response and the effect on performance. Sport. Med..

[17] Jones C.M., Griffiths P.C., Mellalieu S.D. (2017). Training load and fatigue marker associations with injury and illness: A systematic review of longitudinal studies. Sport. Med..

[18] Meeuwisse W. (1994). Assessing causation in sport injury: A multifactorial model. Clin. J. Sport. Med..

[19] Banister E.W., Morton R.H., Fitz-Clarke J. (1992). Dose/response effects of exercise modeled from training: Physical and biochemical measures. Ann. Physiol. Anthropol..

[20] Allen H., Coggan A. (2010). Training and Racing with a Power Meter.

[21] Hulin B.T., Gabbett T.J., Blanch P., Chapman P., Bailey D., Orchard J.W. (2014). Spikes in acute workload are associated with increased injury risk in elite cricket fast bowlers. Br. J. Sport. Med..

[22] Hulin B.T., Gabbett T.J., Caputi P., Lawson D.W., Sampson J.A. (2016). Low chronic workload and the acute:chronic workload ratio are more predictive of injury than between-match recovery time: A two-season prospective cohort study in elite rugby league players. Br. J. Sport. Med..

[23] Gabbett T.J., Hulin B.T., Blanch P., Whiteley R. (2016). High training workloads alone do not cause sports injuries: How you get there is the real issue. Br. J. Sport. Med..

[24] Hellard P., Avalos M., Guimaraes F., Toussaint J.F., Pyne D.B. (2015). Training-related risk of common illnesses in elite swimmers over a 4-yr period. Med. Sci. Sport. Exerc..

[25] Nielsen R.O., Parner E.T., Nohr E.A., Sorensen H., Lind M., Rasmussen S. (2014). Excessive progression in weekly running distance and risk of running-related injuries: An association which varies according to type of injury. J. Orthop. Sport. Phys. Ther..

[26] Coggan A. (2020). Normalized Power, Intensity Factor and Training Stress Score. https://www.trainingpeaks.com/learn/articles/normalized-power-intensity-factor-training-stress/.

[27] Impellizzeri F.M., Tenan M.S., Kempton T., Novak A., Coutts A.J. (2020). Acute:Chronic Workload Ratio: Conceptual Issues and Fundamental Pitfalls. Int. J. Sport. Physiol. Perform..

[28] Gabbett T.J. (2016). The training-injury prevention paradox: Should athletes be training smarter and harder?. Br. J. Sport. Med..

[29] Braun V., Clarke V. (2014). What can “thematic analysis” offer health and wellbeing researchers?. Int. J. Qual. Stud. Health Well-Being.

[30] Zwingenberger S., Valladares R.D., Walther A., Beck H., Stiehler M., Kirschner S., Engelhardt M., Kasten P. (2014). An epidemiological investigation of training and injury patterns in triathletes. J. Sport. Sci..

[31] Kirkland A., Cowley J. (2023). An exploration of context and learning in endurance sports coaching. Front. Sport. Act. Living.

